# Gut Microbiome Analysis and Screening of Lactic Acid Bacteria with Probiotic Potential in Anhui Swine

**DOI:** 10.3390/ani13243812

**Published:** 2023-12-11

**Authors:** Ying Shao, Xiaoyan Wu, Zhaorong Yu, Min Li, Tingting Sheng, Zhenyu Wang, Jian Tu, Xiangjun Song, Kezong Qi

**Affiliations:** 1 Anhui Province Key Laboratory of Veterinary Pathobiology and Disease Control, College of Animal Science and Technology, Anhui Agricultural University, Hefei 230036, China; shaoying@ahau.edu.cn (Y.S.); 15090218106@163.com (X.W.); zhaorongyu@stu.ahau.edu.cn (Z.Y.); 18639204993@163.com (M.L.); shengtingting1030@126.com (T.S.); wangzhenyu@ahau.edu.cn (Z.W.); tujian1980@126.com (J.T.); 2 Anhui Province Engineering Laboratory for Animal Food Quality and Bio-Safety, College of Animal Science and Technology, Anhui Agricultural University, Hefei 230036, China

**Keywords:** HuoShou Black Pig, intestinal contents, diversity of microbiota, high-throughput sequencing, *Lactobacillus*

## Abstract

**Simple Summary:**

In recent years, the increasing harm caused by the excessive use of antibiotics in animal husbandry has drawn attention to the antibacterial properties of lactic acid bacteria. In this study, both 16S rRNA and metagenomic sequencing methods were utilized to analyze the intestinal microbiota of Anhui local pig breeds, and eight strains of lactic acid bacteria were isolated. By evaluating their growth performance and conducting tolerance tests in a simulated intestinal environment, along with assessing autopolymeric hydrophobicity and pathogen inhibition, two dominant strains were selected. This study holds significant guidance for the development, production, and application of lactic acid bacteria resources to local pig breeds.

**Abstract:**

With the widespread promotion of the green feeding concept of “substitution and resistance”, there is a pressing need for alternative products in feed and breeding industries. Employing lactic acid bacteria represents one of the most promising antimicrobial strategies to combat infections caused by pathogenic bacteria. As such, we analyzed the intestinal tract of Anhui local pig breeds, including LiuBai Pig, YueHei Pig, and HuoShou Pig, to determine the composition and diversity of intestinal microbiota using 16S rRNA. Further, the functionality of the pigs’ intestinal microbiota was studied through metagenomic sequencing. This study revealed that lactic acid bacteria were the primary contributors to the functional composition, as determined through a species functional contribution analysis. More specifically, the functional contribution of lactic acid bacteria in the HuoShou Pig group was higher than that of the LiuBai Pig and YueHei Pig. Subsequently, the intestinal contents of the HuoShou Pig group were selected for the screening of the dominant lactic acid bacteria strains. Out of eight strains of lactic acid bacteria, the acid-production capacity, growth curve, and tolerance to a simulated intestinal environment were assessed. Additional assessments included surface hydrophobicity, the self-aggregation capability, co-agglutination of lactic acid bacteria with pathogenic bacteria, and an in vitro bacteriostatic activity assay. *Lactobacillus johnsonii* L5 and *Lactobacillus reuteri* L8 were identified as having a strong overall performance. These findings serve as a theoretical basis for the further development of pig-derived probiotics, thereby promoting the application of lactic acid bacteria to livestock production.

## 1. Introduction

As common probiotics, lactic acid bacteria can maintain the balance of the intestinal microbiota and competitively eliminate pathogens [1,2] as well as improve growth performance, feed conversion efficiency, nutrient utilization, and gut health [3]. They can change the intestinal environment by producing antibacterial substances and reducing the pH value, thus obtaining advantages in intestinal colonization. For example, *Lactobacillus lactis* produces nisin and *Lactobacillus acidophilus* produces bacteriostatic proteins, thus acquiring colonization advantages in the gut. These bacteriocins inhibit and eliminate intestinal pathogens [4,5]. The bacteriocin produced by *Lactobacillus* E50–52 can significantly reduce the number of *Salmonella* in animals [6]. *Lactobacillus rhamnosus* secretes antibacterial substances with inhibitory activities [5] and has a broad-spectrum antibacterial activity against pathogenic bacteria [7]. Scharek et al. [8] found that the intestinal mucosal immune function of weaned piglets was significantly enhanced after feeding them with lactic acid bacteria. Lactic acid bacteria can increase the expression and secretion of β-defensins to prevent the growth and reproduction of pathogenic microorganisms [9]. Studies have demonstrated that the isolated, species-specific strain Pediococcus acidilactici FT28 possessed potential in vitro probiotic properties. More importantly, compared with a piglet control, it showed potential capabilities such as higher nutrient digestibility, hemato-biochemical parameters, and antioxidant status [10]. In another study, lactic acid bacteria isolated from homologous animals played a more stable prebiotic role [11].

In the intestines of pigs, *Firmicutes*, *Bacteroidetes*, and *Proteobacteria* account for approximately 90% of the total intestinal microbiota, but their distribution patterns are different [12]. The main factors causing intestinal microbiota fluctuations may be related to the animal species, diets, and additive contents [12]. The feeding environment is an essential factor affecting the colonization of the intestinal microbiota. In China, a diverse range of pig resources can be observed across the country. In Anhui in particular, we found a variety of high-quality local pig breeds, including the Yuexi Black Pig, HuoShou Black Pig, and six Taihu White Pigs. These breeds display extraordinary traits such as a high tolerance to rough feeding and strong stress resistance. It has been hypothesized that these characteristics might be intricately linked to their gut microbiota [13,14]. However, the specific composition and functions of their gut microbiota remain unexplored.

In summary, this study used high-throughput sequencing to analyze the composition and function of microorganisms in the intestinal tract of Anhui local pig breeds. With traditional isolation and cultivation techniques, we isolated lactic acid bacteria from the intestinal contents of superior breeds of pigs and tested their stress resistance and bacteriostatic ability. The results attained from this study establish a foundation for the future creation of microecological preparations for pigs.

## 2. Materials and Methods

### 2.1. Samples

Intestinal content samples of three pig breeds (Yuexi Black Pig, HuoShou Black Pig, and Taihu LiuBai Pig) were collected, with at least three samples from each pig, totaling 15 samples. The samples were placed in a dry refrigerator after collection and transported to the laboratory. The control strain *Enterococcus faecium* A21 was isolated and preserved by our laboratory. *Escherichia coli* CMCC44102 and *Staphylococcus aureus* ATCC29523 were used as indicator strains.

### 2.2. 16S rRNA Amplicon Sequencing

The DNA extraction was performed according to the QIAamp Fast DNA Stool Mini Kit instructions using the forward primer 338F (5′-ACTCCTACGGGAGGCAGCAGCAGCAG-3′) and the reverse primer 806R (5′-GGACTACHVGGGTWTCTAAT-3′) for the PCR amplification of the V3–V4 variable regions. A QIAquick Gel Extraction Kit was used for PCR product purification and after the amplified product was detected with agarose gel, the cut gel was recovered and purified. The Shanghai Meiji Company was entrusted with the Miseq PE300 platform sequencing.

### 2.3. Metagenomic Sequencing of Intestinal Contents of Local Pigs from the Anhui Province

After extracting genomic DNA, Covaris M220 IIIumina HiSeq sequencing was commissioned using fastp [15] to cut the linker sequences at both ends of the original sequencing data. We used a sliding window with a size of 5 bp starting from both ends of the sequence so that the average mass of bases in the window was more than 20 bp. Finally, sequences with a length above 40 bp and an average base mass above 15 bp were retained. The QC sequence was aligned to the pigs’ reference genomic sequence of Sscrofa11.1 (GCA 000003025.6), retaining the sequence without the alignment. Sequences with a removed host genome were assembled using MEGAHIT, preserving contigs longer than 1000 bp for prediction and gene annotation in open reading frames. Kraken2 [16] was employed to classify the sequence and assembled configs of the deactivated host genome, and Bayesian-based Bracken [17] was used to estimate the abundance of individual species in each sample based on the classification results of kraken2. The gene function was annotated using eggNOG-mapper [18] and the eggNOG database [19]. The system function information, chemical function information, and gene function information were analyzed using the KEGG database. The TPM was calculated using Salmon [20] to estimate the gene abundance.

### 2.4. Isolation and Purification of Lactic Acid Bacteria

We weighed 0.1 g of pig intestinal content, diluted it to an appropriate concentration with sterilized PBS, and evenly coated it onto an MRS solid medium containing 1% CaCO^3^. Subsequently, the sample was placed into a 37 °C incubator and after 24 h, the colony growth and bacterial morphology were observed. Single colonies with neatly edged and milky white calcium-dissolving rings were selected, cultured on MRS agar plates for 24–48 h, continuously purified, and cultured for two to three passages, and then placed in MRS broth for proliferation. After drying, Gram staining was performed; bacteria with purple cells were classified as Gram-positive bacteria, whereas those with red cells were classified as Gram-negative bacteria. The isolated bacteria were extracted following the instructions of the bacterial genome extraction kit and the 16S sequence was amplified. The recovered fragment gel products were subsequently submitted to Beijing Tsingke Biotech Co., Ltd. (Beijing, China). for sequencing. The sequences were then subjected to a BLAST comparison and analysis [21].

### 2.5. Determination of Acid-Production Capacity and Growth Curve

Isolated lactic acid bacteria were activated in MRS broth two to three times, inoculated into 5 mL of an MRS medium at an inoculum volume of 1:100, and incubated at 37 °C for 24 h. The pH value was measured and recorded using a pH meter every 2 h, and a pH value change curve was generated according to the results. The strains with a low pH value in the above acid-production test were selected for activation and a seeding volume of 1:100 was inserted into the MRS liquid medium and incubated at 37 °C for 24 h. Subsequently, the OD_600nm_ value was measured every 2 h and the absorbance was calibrated using blank MRS as the control; the growth curve was generated with the measured OD value as the ordinate.

### 2.6. Acid-Resistance Determination

We adjusted the MRS broth medium with hydrochloric acid to pH 2.0 and pH 3.0, autoclaved it, and set it aside. After activating the screened strain two to three times, it was inoculated into 5 mL of liquid MRS medium at 1% and incubated in a 37 °C incubator for 24 h. We set the MRS broth with the unadjusted pH as a negative control. Absorbance at OD_600nm_ was determined after 0 and 4 h, and the diluted sample was evenly coated on the MRS plates. The survival rate was calculated according to the plate-counting method using the following equation:

Survival (%) = N2/N1 × 100%

where N1 is the number of viable bacteria (cfu/mL) cultured for 0 h in the MRS liquid medium at pH 2.0 and pH 3.0, respectively, and N2 is the number of viable bacteria after 4 h of culture.

### 2.7. Determination of Bile Salt Resistance

The strain was placed into 5 mL of an MRS broth medium at an inoculation volume of 1:100. After standing at 37 °C for 24 h, the bile salt content of the MRS broth was adjusted to 0.1% and 0.3% using porcine bile salt and the MRS broth without bile salt regulation was set as a negative control. Samples were taken after standing the culture for 0 and 4 h, and the survival rate was calculated using the plate-counting method [21].

### 2.8. Trypsin Tolerance Measurement

We adjusted the concentration of the MRS liquid medium with a trypsin solution to 1 mg/mL, pipetted 1 mL of the bacterial solution, centrifuged it at 8000 r/min for 3 min, and carefully washed it two to three times with a PBS buffer. The bacterial cells were suspended in 1 mL PBS, inoculated into 5 mL of MRS broth at 1%, and cultured at 37 °C for 24 h; MRS broth without trypsin regulation was used as a negative control. Samples were taken and the MRS plates were evenly coated after standing the culture for 0 and 4 h. The survival rate was calculated using the plate-counting method [22].

### 2.9. Surface Hydrophobicity

Referring to the microbial adhesive hydrocarbon compound method (BATH) [23], the screened lactic acid bacteria were activated and centrifuged at 8000 r/min for 10 min, then the supernatant was removed. The resuspended bacteria were washed with a PBS buffer and the operation was repeated two to three times. Subsequently, we resuspended the bacterial cells in 0.1 M of a KNO_3_ solution and adjusted its absorbance to OD_600nm_ = 0.5 ± 0.02 (A0) under OD_600nm_. We then added 1 mL of xylene and 3 mL of the KNO_3_ resuspension solution and allowed the mixture stand for 15 min. After shaking for 60 s and standing for 15 min until the solution showed aqueous phase stratification, the aqueous phase was slowly absorbed to determine absorbance (A1) at OD_600nm_. Hydrophobicity was determined as follows:

Hydrophobicity (%) = (1 − A1/A0) × 100%.

### 2.10. Self-Aggregation Capability

According to the method of Xu et al. [24], we placed 2 mL of bacterial suspension in a centrifuge tube, followed by shaking for 15 s and standing at 37 °C for 2 h. Subsequently, 1 mL of the supernatant was removed, and absorbance (B1) at OD_600nm_ was determined. Self-aggregation was determined using the following equation:

Self-aggregation (%) = (1 − B_1_/B_0_) × 100%

### 2.11. Co-Agglutination of Lactic Acid Bacteria with Pathogenic Bacteria

Referring to the steps described in Collado et al. [25], the lactic acid bacteria were obtained by preliminary screening and the concentration of the bacterial solution was adjusted to 1.0 × 10^8^ (cfu/mL). Subsequently, we aspirated 1.5 mL of lactic acid bacteria and mixed it with a causative bacteria suspension, then added 3 mL of the PBS dilution to mix. After the three groups of bacteria were thoroughly shaken for 20 s, they were placed in a temperature box at 37 °C and incubated for 2 h. We then slowly pipetted 500 μL of the supernatant and determined the absorbance values of C1 (mixed bacterial solution), C2 (lactic acid bacteria single bacterial solution), and C3 (pathogenic bacteria single bacterial solution) at OD_600nm_ [26]. Co-agglutination was calculated using the following equation:

Co-aggregation (%) = [1 − C1/(C2 + C3)] × 100%.

### 2.12. In Vitro Bacteriostatic Activity Assay

The antibacterial activity of lactic acid bacteria against the index bacteria *Escherichia coli* CMCC44102 and *Staphylococcus aureus* ATCC25923 was determined by agar perforation diffusion. The isolated lactic acid bacteria were inoculated at 1:100 into the MRS broth; after 24 h, the mixture was centrifuged at 6000× *g* for 15 min and the supernatant was removed. The LB solid medium was prepared with 0.1% indicator bacteria; holes were punched using an 8 mm sterile punch and a fresh culture solution as well as the supernatant of the 100 μL isolate were added. The plate was placed into an incubator at 37 °C and cultured; subsequently, we determined the diameter of the inhibition circle.

### 2.13. Determination of Inhibition of Pathogenic Bacteria

After standing at 37 °C for 24 h in MRS broth, *L. johnsonii* L5 and *L. reuteri* L8 were centrifuged at 6000 r/min for 10 min. The supernatant was collected and filtered. After activation, *E. coli* CMCC44102 was inoculated at a 1% ratio to the supernatant of *L. johnsonii* L5 and *L. reuteri* L8; the LB medium was set as the control group. After 16 h, 10 μL of the culture was dropped onto the center of the LB solid medium and incubated at 37 °C for 6 h. Subsequently, the movement diameter of *E. coli* was measured and the experiment was repeated three times.

### 2.14. Data Analysis

SPSS 26 data analysis software was used for the data processing and significance analysis. The test results were expressed as the mean ± standard deviation. Different lower case letters indicated a significant difference (*p* < 0.05) and the same letter or no letter indicated no significant difference (*p* > 0.05).

## 3. Results

### 3.1. Analysis of the Intestinal Microbiota Composition of Anhui Local-Breed Pigs

After quality control and filtration, a total of 1,014,440 valid sequences were obtained from 15 intestinal content samples. Each sample contained an average of 67,629 reads with an average sequence length of 415 bp.

**Figure 1:**
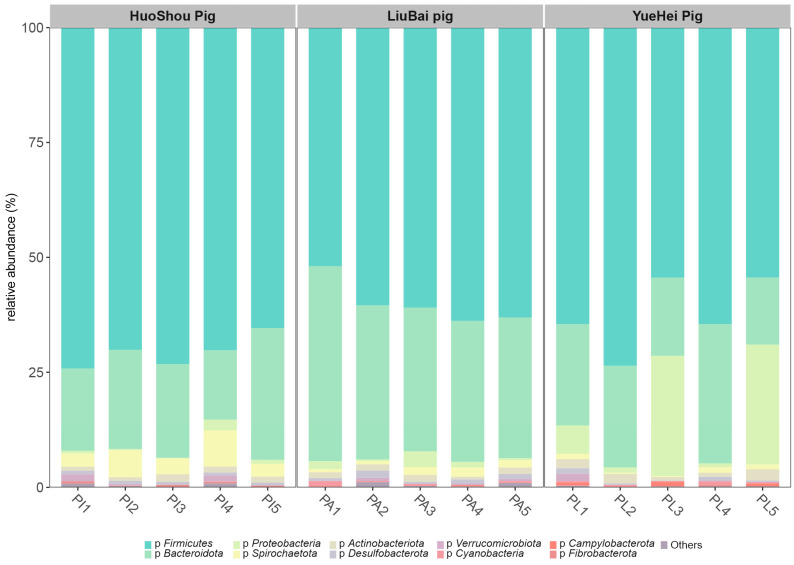
Histogram of relative species abundances of pig intestinal microbiota at the phylum level. The *x* axis is the name of each group of samples and the *y* axis is the relative percentage of the amplitude of bacteria at the phylum level.

At the phylum level, a total of 17 bacterial phyla were identified in the HuoShou Pig group, 20 bacterial phyla were identified in the LiuBai Pig group, and 22 bacterial phyla were identified in the YueHei Pig group. Through species comparison and annotation, the distribution of bacteria in the intestinal contents of the three groups was ascertained and is shown in Figure 1. The five phyla with relative species abundances above 1% were *Firmicutes*, *Bacteroidetes*, *Proteobacteria*, *Spirochaetes*, and *Actinobacteria*.

The abundance of *Firmicutes* and *Spirochaetes* in the HuoShou Pig group was higher than in the LiuBai Pig and YueHei Pig groups. *Bacteroidetes* in the LiuBai Pig group showed a higher abundance, and the abundance of *Proteobacteria* in the YueHei Pig group was significantly higher than in the HuoShou Pig and LiuBai Pig groups. *Firmicutes* and *Bacteroidetes* were the most abundant phyla common to the three groups of pigs, which was consistent with the results of previous studies (Figure 1).

**Figure 2:**
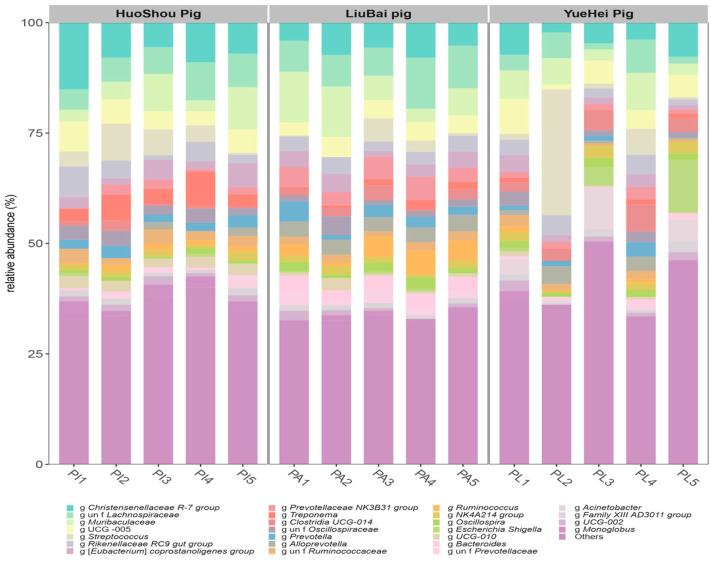
Histogram of relative species abundances of pig intestinal microbiota at the genus level. The *x* axis is the name of each group of samples and the *y* axis is the relative percentage of the amplitude of bacteria at the phylum level.

As shown in Figure 2, a total of 260 bacterial genera were identified in the HuoShou Pig group, 245 in the LiuBai Pig group, and 370 in the YueHei Pig group. The core genera were similar among the three groups and were the *Christensenellaceae R-7 group*, *Lachnospiraceae*, *Muribaculaceae*, *UCG-005*, *Streptococcus*, *Rikenellaceae RC9 gut group*, *Clostridia UCG-014*, the (*Eubacterium*) *coprostanoligenes group*, the *Prevotellaceae NK3B31 group*, and *Treponema*.

**Figure 3:**
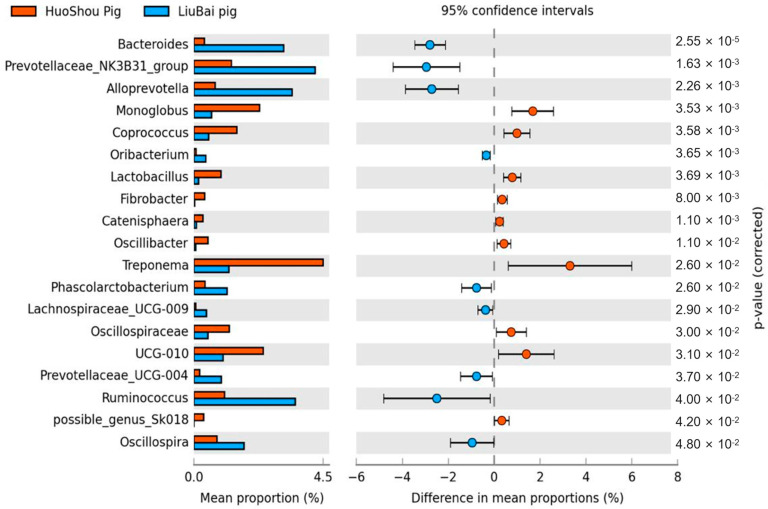
Differences in the abundances of the main genera in the HuoShou Pig and LiuBai Pig groups. Positive differences indicate that genera were more abundant in HuoShou Pigs and negative differences indicate a higher abundance in LiuBai Pigs.

The intestinal microbiota of the pigs of the three groups was compared, in pairs, with differences in the genera, according to the *t*-test. There were 19 genera with significant differences in abundance in the intestinal microbiota of pigs in the HuoShou Pig and LiuBai Pig groups. The genera with higher abundances in the HuoShou Pig group included *Monoglobus*, *Coprococcus*, *Lactobacillus*, *Fibrobacter*, *Catenisphaera*, *Oscillibacer*, *Treponema*, *Oscillospiraceae*, *UCG-10*, and *possible genus Sk018*. In the LiuBai Pig group, *Bacteroides*, the *Prevotellaceae NK3B31 group*, *Phascolarctobacterium, Lachnospiraceae UCG-009*, *Alloprevotella*, *Oribacterium*, *Prevotellaceae UCG-004*, *Ruminococcus*, and *Oscillospira* were the most abundant (Figure 3).

**Figure 4:**
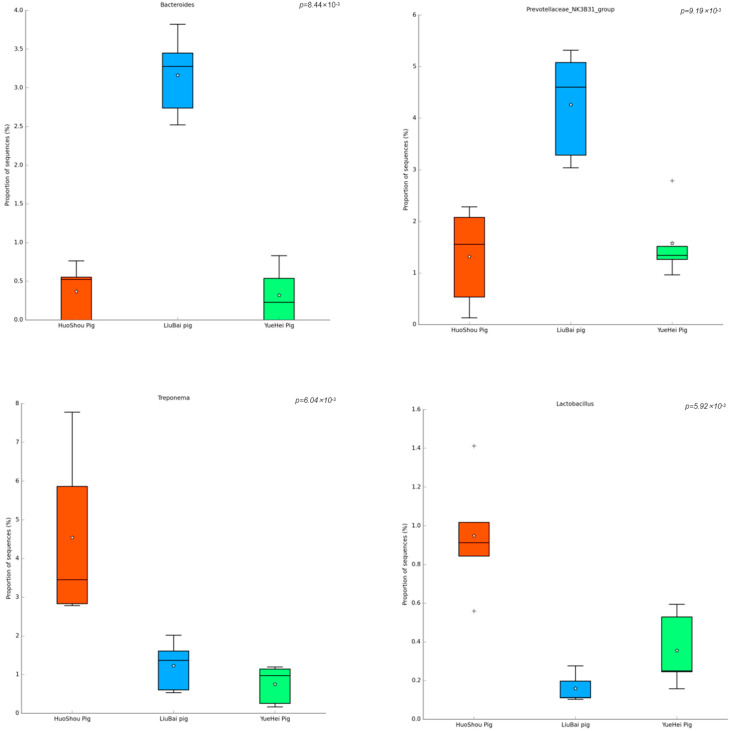
Differences in the bacterial genera of the intestinal contents from the three pig groups.

When comparing the intestinal microbiota of the three groups of pigs at the genus level, the most abundant genera were *Bacteroides*, *Prevotellaceae NK3B31 group*, *Treponema*, and *Lactobacillus*. As shown in Figure 4, the richness of *Bacteroides* and *Prevotellaceae NK3B31 group* in the LiuBai Pig group was significantly higher than the HuoShou Pig group and the YueHei Pig group. The richness of *Treponema* and *Lactobacillus* in the HuoShou Pig group was significantly higher than the LiuBai Pig and YueHei Pig groups.

### 3.2. Functional Analysis of the Intestinal Microbiota of Anhui Local-Breed Pigs

#### 3.2.1. Raw Data Processing

A total of 434,461,566 raw reads were obtained for sequencing. The average sequence had 48,273,507 reads and the overall and average data volume after quality control were 417,479,600 and 46,386,622 reads, respectively. The effective data rate after quality control was 96.09% (Table 1). As shown in Table 2, a total of 3,002,533 contigs were obtained after assembling the quality control sequences, with an average sequence length of 1255 bp and a maximum length of 461,768 bp, with 1432 bp for N50 and 581 bp for N90.

#### 3.2.2. COG Function Analysis of the Porcine Intestinal Microbiota

**Figure 5:**
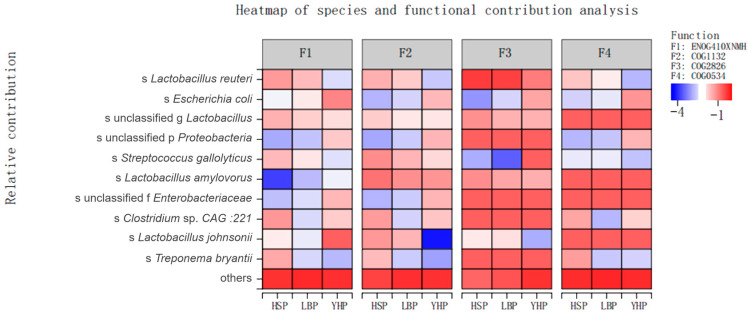
Analysis of the functional contribution at species level. F1: ENOG410XNMH (histidine kinase and signaling); F2: COG1132 (transporter, defense mechanism, cellular process, and signaling); F3: COG2826 (transposase and putative membrane protein); F4: COG0534 (multidrug efflux pump and leucine-responsive regulatory protein).

Lactic acid bacteria provided the main functional contribution. The functional annotation of *Lactobacillus amylovorus* was mainly the COG1132 ABC transporter (transporter, defense mechanism, cellular process, and signaling). For *Lactobacillus reuteri*, the function of the main annotation was COG2826 transposase and ENOG410XNMH (histidine kinase and signaling). For unclassified Lactobacillus, the function of the main annotation was COG2826 transposase and COG0634. The contribution of lactic acid bacteria from the HuoShou Pig group was generally higher than the other two varieties. The contribution of lactic acid bacteria in the YueHei Pig group was concentrated in COG2826 transposase, which was hardly reflected in other functions, while lactic acid bacteria in the HuoShou Pig group provided the main function, as shown in Figure 5.

### 3.3. Functional Analysis of Porcine Intestinal Lactic Acid Bacteria

**Figure 6:**
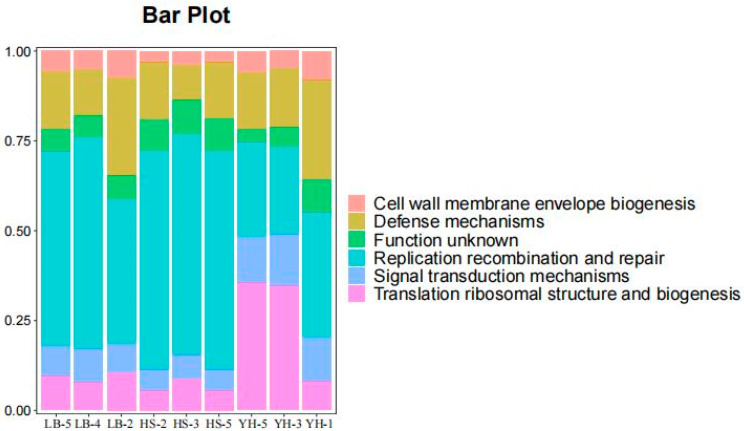
Functional contribution analysis of lactic acid bacteria.

A species annotation of the lactic acid bacteria yielded three genera; namely, *Lactobacillus*, *Pediococcus*, and *Sharpea*. The COG function for the annotated lactic acid bacteria included cell wall membrane envelope biogenesis, defense mechanisms, replication recombination and repair, signal transduction mechanisms, translation ribosomal structure, and biogenesis as shown in Figure 6.

### 3.4. Isolation and Screening of Porcine Intestinal Lactic Acid Bacteria

From the intestinal contents of the HuoShou Black Pig group, 73 lactic acid bacteria (S1) strains, characterized by a milky white color, clear calcium solubility rings, and neat edges, were isolated. The 16S rRNA gene sequences were amplified and followed by a BLAST analysis, which led to the isolation and biochemical identification of eight Lactobacillus strains. These eight strains of Lactobacillus underwent biochemical identification, the results of which are presented in Table 3.

### 3.5. Determination of Acid-Production Capacity and Growth Curve

**Figure 7:**
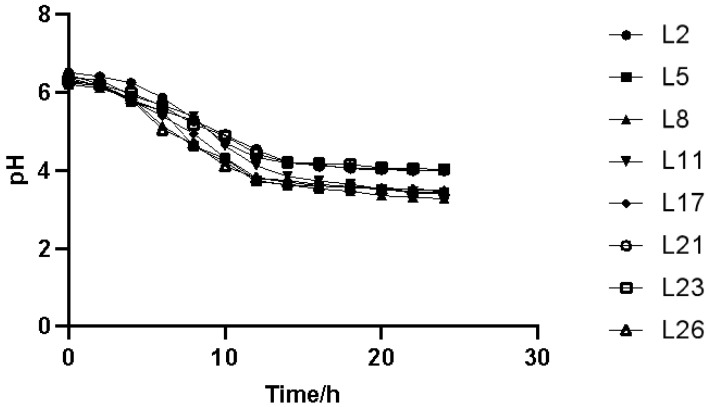
Acid-producing capacity curves of some lactic acid bacteria.

**Figure 8:**
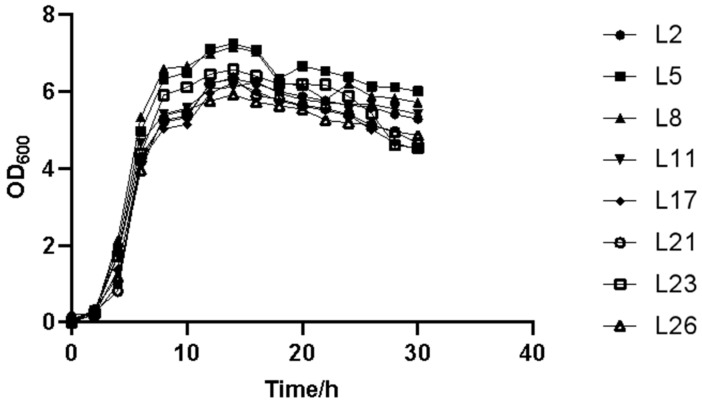
Partial growth curves of lactic acid bacteria.

The acid-production curves of the eight lactic acid bacteria are shown in Figure 7. All bacteria showed excellent acid-production performance; at approximately 12 h, the pH values of L5, L8, and L11 dropped to less than 4.0, indicating a high acid production. As shown in Figure 8, L2, L5, and L8 entered the logarithmic growth phase at approximately 4 h, whereas the remaining bacteria gradually entered the logarithmic growth state at 6 h. In the period of 10–15 h, L5, L8, and L23 lactic acid bacteria showed a higher biomass. At about 16 h, the eight lactic acid bacteria basically reached a stable growth state and the OD value change fluctuation was small, indicating good growth characteristics.

### 3.6. Endurance Measurement Results

As shown in Table 4, at pH 3, the survival rates for all strains (except L23) were above 65%. At pH 2.0, the survival rates of L5, L8, L11, and L21 reached 60%. At a bile salt concentration of 0.1%, the survival rates of L5, L11, L26, and L8 exceeded 60% and at a bile salt concentration of 0.3%, L5 and L8 still maintained half the survival rate. At a trypsin concentration of 1.0%, the viable numbers of L5 and L8 were greater than 1 × 10^8^ cfu/mL and the survival rate exceeded 50%. In summary, L5, L8, and L11 showed a higher tolerance to these conditions.

### 3.7. Surface Hydrophobicity and Self-Polymerization Ability

The self-polymerization capacity of L2, L5, L8, and L17 was above 25%. The self-polymerization capacity of L8 reached 42.97%, which was significantly higher than that of the other strains (*p* < 0.05). The surface hydrophobicity of L2, L5, and L8 reached more than 70%, with that of L5 reaching 76.70%, which was significantly higher than that of the other strains (*p* < 0.05). The hydrophobicity and self-polymerization ability values of the eight strains are shown in Table 5.

### 3.8. Co-Agglutination with Pathogenic Bacteria

**Figure 9:**
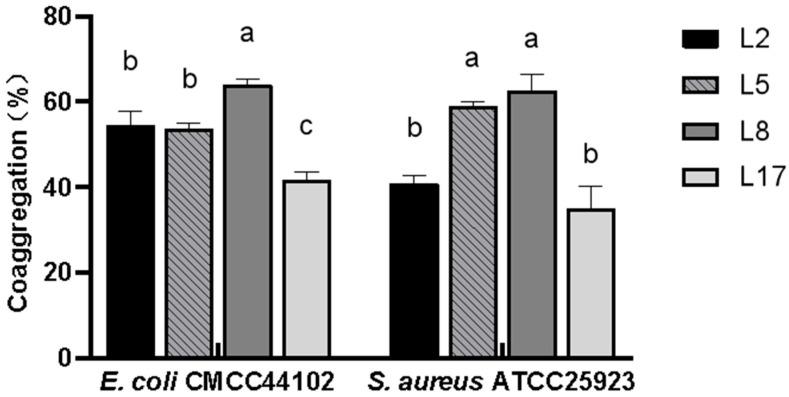
Co-agglutination abilities of some lactic acid bacteria against pathogenic bacteria. Mean ± SD was used for data recording. Letters (such as “a”, “b”, “c”, etc.) following the numerical values represent statistical significance levels, with different letters indicating statistical significance (*p* < 0.05). The same letter denotes no statistically significant difference (*p* > 0.05).

Co-agglutination experiments were performed for strains L2, L5, L8, and L17 with the pathogenic bacteria *E. coli* CMCC44102 and *S. aureus* ATCC25923 are shown in Figure 9. The co-agglutination rate of L8 against *E. coli* CMCC44102 was significantly higher than the other three lactic acid bacteria, whereas the co-agglutination rate of L17 against *E. coli* CMCC44102 was significantly lower than the other three groups. In the co-agglutination test with *S. aureus* ATCC25923, the co-agglutination capacity of L5 and L8 was significantly higher than L2 and L17. The co-agglutination rate of L5 against *S. aureus* ATCC25923 was higher than against *E. coli* CMCC44102.

### 3.9. In Vitro Bacteriostatic Test

When testing the inhibition of *E. coli* CMCC44102, the inhibition diameters for strains L5, L8, and L17 reached more than 16 mm; that for L8 was as high as 17.67 mm, which was significantly different from those observed for the other lactic acid bacteria (*p* < 0.05). In the inhibition diameter of the supernatant, there was no significant difference when comparing the antibacterial effect of the fermentation broth supernatant and the strain’s stock solution. When testing the inhibition of *S. aureus* ATCC29523 via the agar diffusion test, the inhibition diameter of the stock solutions and supernatants of L2, L5, L8, and L17 lactic acid bacteria reached more than 16 mm, whereas that of L11 and L21 was below 14 mm. The results for the eight bacterial strains are shown in Table 6.

### 3.10. Effects of L. johnsonii L5 and L. reuteri L8 on the Motility of E. coli CMCC44102

**Figure 10:**
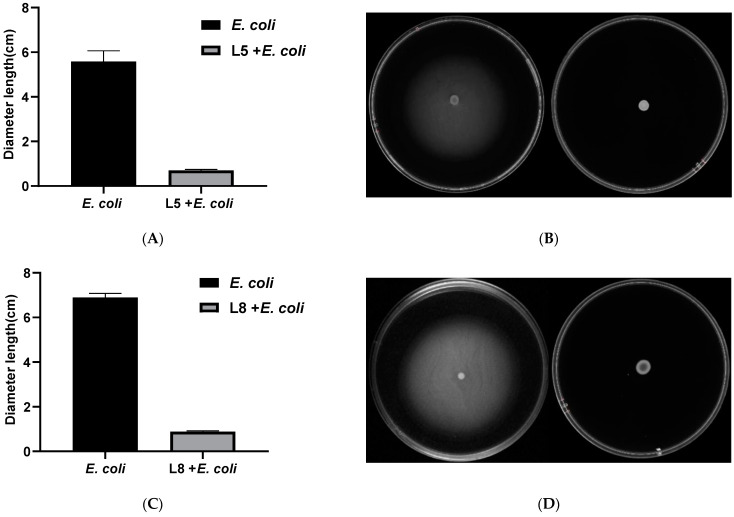
Effects of *Lactobacillus johnsonii* L5 and *Lactobacillus reuteri* L8 on the motility of *Escherichia coli* 44102. (**A**,**C**) Effects of *L. johnsonii* L5 and *L. reuteri* L8 supernatants on CMCC44102 motility expressed as mean ± SEM. (**B**,**D**) Motility determination images of CMCC44102 and the control group treated with *L. johnsonii* L5 and *L. reuteri* L8 supernatant.

As shown in Figure 10, the *L. johnsonii* L5 and *L. reuteri* L8 cell-free fermentation supernatants significantly (*p* < 0.05) reduced the motility of *E. coli* CMCC44102. After treatment with the *L. johnsonii* L5 cell-free fermentation supernatant, the motion diameter of *E. coli* CMCC44102 decreased to 0.4255 ± 0.0813 cm, whereas the motion diameter of the untreated control group was 4.462 ± 0.1302 cm. The motion diameter of *E. coli* decreased to 0.4937 ± 0.0655 cm after the *L. reuteri* L8 cell-free fermentation supernatant treatment compared with 4.913 ± 0.1057 cm in the control group. The *L. Johnsonii* L5 and *L. reuteri* L8 cell-free fermentation supernatants significantly inhibited the motility of *E. coli* CMCC44102.

## 4. Discussion

In this study, the intestinal contents of Anhui local pigs of different breeds were analyzed using 16S rDNA sequencing to determine the composition and diversity of the intestinal bacteria. The main functions included cell wall membrane envelope biogenesis, defense mechanisms, replication recombination and repair, signal transduction mechanisms, and the translation ribosomal structure and biogenesis. The contribution of lactic acid bacteria in the YueHei Pig group was almost not reflected, whereas the lactic acid bacteria in the HuoShou Pig group contributed most significantly to the functional annotation.

As an important probiotic, lactic acid bacteria must overcome the adverse environment of the gastrointestinal tract when entering the body via ingestion to ensure survival. The hyperpermeable environment caused by high levels of gastric acid and bile salts in the gastrointestinal tract is the primary problem faced by bacteria in this habitat. A pH of 3.0 is one of the most important criteria for probiotic tolerance and screening [27]. The lactic acid bacteria screened in this trial had a low survival rate at pH 2.0, but the survival rate of *L. reuteri* L8 and *L. johnsonii* L5 at pH 2.0 reached 60%, showing strong acid resistance. Studies have shown that most lactic acid bacteria exhibit good viability at pH 3.0 and low viability at pH 2.0 [28].

The tolerance of lactic acid bacteria to small intestinal transport is critical for their colonization and metabolism. The bile salt concentration is one of the important factors affecting the colonization of microorganisms in the intestine and most lactobacilli play a probiotic role due to their bile salt tolerance and stable presence in the intestine [29,30]. The greater the bile salt tolerance of *Lactobacillus*, the more conducive it is to colonizing the intestine and exerting physiological activity. Lactic acid bacteria from different sources have different bile salt tolerances [31]. Zhang Li et al. [32], regarding the tolerance of *Lactobacillus* bile salts isolated from yak milk, found that the tolerance of different strains to different bile salts increased over time at a concentration of 0.3% bile salts. In addition, animal-derived *Lactobacillus* bile salts are better tolerated than those from other sources; possibly, animal-derived strains that have adapted to the gastrointestinal environment exhibit better probiotic properties [33]. In this study, lactic acid bacteria isolated from the intestinal contents of HuoShou pigs were subjected to different bile salt concentrations and a trypsin tolerance assay. *Lactobacillus johnsonii* L5 and *L. reuteri* L8 retained a viable bacterial count of 1.0 × 10^8^ cfu/mL at a bile salt concentration of 0.3% and a trypsin level of 1.0%, showing a higher tolerance to these conditions.

Due to some differences among strains, the adhesion performance of lactic acid bacteria in the intestine also varies [34]. In this study, the adhesion capacity was determined using the BATH method and a self-polymerization test; strains L2, L5, L8, and L17 showed a good performance. The hydrophobicity of L2, L5, and L8 was as high as 70%. When co-agglutinated with the pathogenic bacteria *E. coli* CMCC44102 and *S. aureus* ATCC25923, *L. johnsonii* L5 and *L. reuteri* L8 had a higher agglutination effect and co-agglutination with *S. aureus* ATCC25923 was more obvious. The combination of *L. johnsonii* L5 and *L. reuteri* L8 showed a more significant hydrophobicity and agglutination of pathogenic bacteria. Highly hydrophobic lactic acid bacteria exhibit a higher affinity with the intestinal mucosa, thus rendering a greater prospect of colonization, a quintessential prerequisite for lactic acid bacteria to function as probiotics. Furthermore, the enhanced agglutination of pathogenic bacteria is advantageous in thwarting the diffusion and subsequent infections of pathogenic microorganisms within the intestinal tract [35]. Safety is a prerequisite for the clinical use of probiotics. During the screening process, the strain should be isolated from healthy animals [36]. Studies have shown that some lactobacilli can secrete bacteriocins that inhibit the growth of gastrointestinal pathogens [37]. In this test, *E. coli* CMCC44102 and *S. aureus* ATCC25923 were selected as indicator strains and the isolated lactic acid bacteria were subjected to the Oxford cup antibacterial test. The inhibition diameters of strains L2, L5, L8, and L17 reached more than 16 cm, indicating a strong bacteriostatic activity.

In summary, combined with the traditional isolation and culturing, two strains of *Lactobacillus johnsonii* with strong stress resistance and probiotic effects were successfully screened from the intestinal contents of the HuoShou Pig. This has important guiding significance for the development and production of lactic acid bacteria resources with local-breed pigs.

## 5. Conclusions

In this study, high-throughput sequencing technology was used to systematically analyze the composition and functional characteristics of the intestinal microbiota of three local pigs. Combined with traditional isolates, *L. Johnsonii* L5 and *L. reuteri* L8 were screened, with a strong stress resistance and probiotic effect. This provides a theoretical basis for the further development of porcine probiotics and the effects on animals as feed additives can be further studied.

## Figures and Tables

**Table 1:** Basic sequence data.

Sample ID	Raw Reads	Clean Reads	Q20 %	Q30 %	GC %	Duplicate %
HS-2	48,343,266	46,295,856	98.54	94.62	43.40	10.11
HS-5	48,798,380	46,749,380	98.56	94.68	42.40	10.58
HS-3	44,748,646	43,054,834	98.63	94.88	41.42	9.61
LB-4	49,869,760	47,902,752	98.69	95.12	45.71	10.30
LB-2	52,068,592	50,317,040	98.76	95.37	49.00	10.40
LB-5	45,744,046	43,907,488	98.61	94.90	46.82	9.93
YH-3	46,639,660	44,777,718	98.66	95.06	47.69	9.88
YH-5	47,181,778	45,330,722	98.69	95.15	48.60	10.20
YH-1	51,067,438	49,143,810	98.76	95.33	46.27	10.89

Note: Sample ID stands for sample name; Raw reads represent the original sequence of deplaning; Clean reads are the effective sequences obtained by filtering; Q (20%) and Q (30%) represent the percentages of clean reads with sequencing error rates less than 0.01 (mass value > 20) and 0.001 (mass value > 30); GC (%) represents the percentage of the GC base content in clean reads; Duplicate (%) indicates the same sequence after quality control.

**Table 2:** Basic data after quality control.

Sample ID	Contigs	Average (bp)	Max (bp)	N50	N90
HS-2	333,797	1273.38	194,692	1452	586
HS-3	207,370	1434.83	461,768	1861	596
HS-5	292,105	1226.07	283,922	1355	578
LB-2	369,417	1328.48	380,063	1602	589
LB-4	385,374	1229.60	312,006	1354	582
LB-5	392,067	1208.52	184,693	1316	579
YH-1	453,318	1193.34	355,447	1295	576
YH-3	293,544	1217.91	197,853	1366	575
YH-5	274,541	1184.62	176,139	1291	570

Note: Sample ID stands for sample name; Contigs indicate the number of contigs sequences; Average indicates the average sequence length of contigs; N50 (N90) indicates that the contig sequences were sorted by length and the length values of each contig sequence were scanned one by one from large to small for the accumulative sum. When the accumulative value exceeded 50% (90%) of the total length of all contigs for the first time, the length value of the scanned sequence was N50 (N90); Max represents the sequence length of the longest contig.

**Table 3:** Biochemical identification results of some lactic acid bacteria.

Strains	Mannitol	Maltose	Sucrose	Lactose	Cellobiose	Inulin	Esculin	Sorbitol	Salicylin	Raffinose
L2	+	+	+	+	+	+	+	+	−	−
L5	+	+	+	+	+	+	−	+	+	+
L8	−	+	+	+	+	+	+	−	+	+
L11	+	+	+	+	+	+	+	−	+	+
L17	−	+	+	+	+	+	+	−	+	−
L21	+	+	+	+	−	+	+	+	+	−
L23	+	+	+	+	+	+	−	−	+	+
L26	−	+	+	+	+	+	+	−	+	+

Notes: “+” indicates a positive fermentation result; “−” indicates a negative fermentation result.

**Table 4:** Effects of different pH values, bile salt concentrations, and protease levels on the survival rates of lactic acid bacteria.

Strains	Control (cfu/mL)	Survival Rate (%)				
		pH		Bile Salt Concentration (%)		Trypsin Concentration (%)
		pH 2.0	pH 3.0	0.10%	0.30%	1.0%
L2	4.32 × 10^7^	48.3 ± 1.04 ^d^	67.37 ± 1.8 ^d^	51.17 ± 0.45 ^de^	24.3 ± 0.89 ^c^	28.48 ± 2.21 ^b^
L26	2.93 × 10^8^	55.13 ± 1.46 ^c^	75 ± 1.56 ^c^	61.57 ± 2.18 ^c^	32.2 ± 1.31 ^b^	18.4 ± 4.69 ^cd^
L5	2.64 × 10^8^	65.53 ± 1.92 ^a^	85.4 ± 1.71 ^a^	73.1 ± 2.85 ^a^	53.3 ± 0.89 ^a^	54.73 ± 3.37 ^a^
L8	2.27 × 10^8^	63.23 ± 0.2 ^ab^	83.27 ± 0.12 ^ab^	62.37 ± 0.96 ^bc^	51.4 ± 1.41 ^a^	53.33 ± 3.21 ^a^
L11	1.82 × 10^8^	63.51 ± 0.25 ^ab^	73.23 ± 0.25 ^c^	64.77 ± 1.53 ^b^	36.73 ± 3.21 ^b^	17 ± 0.63 ^cd^
L17	2.86 × 10^7^	56.13 ± 0.86 ^c^	68.8 ± 0.56 ^d^	38.63 ± 0.84 ^f^	17.8 ± 0.92 ^de^	23.33 ± 4.16 ^bc^
L21	1.75 × 10^8^	61.67 ± 1.39 ^b^	81.67 ± 1.39 ^b^	50.5 ± 0.92 ^e^	21.67 ± 6.75 ^cd^	18.33 ± 6.03 ^cd^
L23	3.04 × 10^7^	15.57 ± 1.81 ^e^	55.57 ± 1.81 ^e^	53.6 ± 0.36 ^d^	15.4 ± 1.65 ^e^	15.33 ± 1.53 ^d^

Note: Mean ± SD was used for data recording. Letters (such as “a”, “b”, “c”, etc.) following the numerical values represent different statistical significance levels, with different letters indicating statistical significance (*p* < 0.05). The same letter denotes no statistically significant difference (*p* > 0.05).

**Table 5:** Self-polymerization ability and surface hydrophobicity of different lactic acid bacteria.

Strain	Self-Polymerization Ability (%)	Surface Hydrophobicity (%)
L2	31.63 ± 2.65 ^b^	72.9 ± 2.76 ^ab^
L26	23.7 ± 1.91 ^cd^	63.37 ± 1.62 ^c^
L5	33.37 ± 1.04 ^b^	76.7 ± 4.77 ^a^
L8	42.97 ± 2.31 ^a^	71.61 ± 2.29 ^ab^
L11	21.27 ± 7.65 ^d^	51.27 ± 6.05 ^de^
L17	27.7 ± 0.6 ^bc^	47.1 ± 0.6 ^f^
L21	10.53 ± 1.39 ^e^	56.93 ± 4.57 ^d^
L23	9.1 ± 1.75 ^e^	66.3 ± 3.41 ^bc^

Note: Mean ± SD was used for data recording. Letters (such as “a”, “b”, “c”, etc.) following the numerical values represent statistical significance levels, with different letters indicating statistical significance (*p* < 0.05). The same letter denotes no statistically significant difference (*p* > 0.05).

**Table 6:** Bacteriostatic effects of different lactic acid bacteria on pathogenic bacteria.

Strain	Inhibition Diameter (mm)			
	*E. coli* CMCC44102		*S. aureus* ATCC25923	
	Strain’s Stock Solution	Supernatant	Strain’s Stock Solution	Supernatant
L2	13.8 ± 0.36 ^d^	14.63 ± 0.12 ^de^	16.17 ± 0.76 ^c^	16.37 ± 0.21 ^bc^
L26	14.67 ± 0.4 ^d^	14.23 ± 0.65 ^de^	14.73 ± 0.76 ^d^	15.5 ± 1.25 ^c^
L5	16.4 ± 0.46 ^b^	16.43 ± 0.15 ^b^	18.1 ± 0.44 ^a^	17.4 ± 1.08 ^ab^
L8	17.67 ± 0.31 ^a^	17.77 ± 0.25 ^a^	17.8 ± 0.2 ^ab^	18.13 ± 0.61 ^a^
L11	15.73 ± 0.45 ^bc^	15.93 ± 0.55 ^bc^	12.53 ± 0.21 ^e^	12.8 ± 0.78 ^d^
L17	16.6 ± 1.18 ^b^	16.77 ± 0.98 ^b^	16.67 ± 0.21 ^bc^	17.53 ± 0.38 ^ab^
L21	13.8 ± 0.1 ^d^	14.13 ± 0.59 ^e^	13.33 ± 1.6 ^e^	13.4 ± 1.35 ^d^
L23	14.87 ± 0.74 ^cd^	15.2 ± 0.56 ^cd^	15.5 ± 0.26 ^cd^	15.83 ± 0.71 ^bc^

Note: Mean ± SD was used for data recording. Letters (such as “a”, “b”, “c”, etc.) following the numerical values represent statistical significance levels, with different letters indicating statistical significance (*p* < 0.05). The same letter denotes no statistically significant difference (*p* > 0.05).

## Data Availability

Data are contained within the article.
